# Socioeconomic Patterns of Twitter User Activity

**DOI:** 10.3390/e23060780

**Published:** 2021-06-19

**Authors:** Jacob Levy Abitbol, Alfredo J. Morales

**Affiliations:** 1 GRYZZLY SAS, 69003 Lyon, France; jacob@gryzzly.io; 2 MIT Media Lab, Cambridge, MA 02139, USA

**Keywords:** human behavior, socioeconomic status, data analysis, social media

## Abstract

Stratifying behaviors based on demographics and socioeconomic status is crucial for political and economic planning. Traditional methods to gather income and demographic information, like national censuses, require costly large-scale surveys both in terms of the financial and the organizational resources needed for their successful collection. In this study, we use data from social media to expose how behavioral patterns in different socioeconomic groups can be used to infer an individual’s income. In particular, we look at the way people explore cities and use topics of conversation online as a means of inferring individual socioeconomic status. Privacy is preserved by using anonymized data, and abstracting human mobility and online conversation topics as aggregated high-dimensional vectors. We show that mobility and hashtag activity are good predictors of income and that the highest and lowest socioeconomic quantiles have the most differentiated behavior across groups.

## 1. Introduction

Historically, governments have quantified natural and societal systems in order to outline and validate public policies, and to organize their territory [1,2,3]. Having socioeconomic data to guide the design of these policies is nevertheless crucial. However, gathering such information can represent a challenge for governments and corporations given the costly efforts associated to the deployment of large-scale national surveys. This is especially the case in developing countries, whose governments may lack the resources needed for completing such endeavors. The recent access to datasets collected from social media and other electronic platforms has enabled the direct observation of individuals and social behaviors [4]. These new sources of data, when properly mined through efficient algorithmics, can provide researchers with an in-depth view of social processes hard to obtain otherwise.

Data obtained from social media enabled an unprecedented analysis of the complexity of societies [4]. Recent studies have shown patterns of social behaviors across multiple scales of observation, ranging from individual preferences up to the structure and dynamics of self-organized groups and collectives [5,6,7]. Example applications of these analyses include the analysis of stock market variations based on collective sentiment analysis [8], the prediction of electoral results [9], the political polarization of societies [10], and the relationship between health and shopping preferences [11]. These types of studies have only become more prevalent with the rising ease of access to geolocated data, enabling the modeling and prediction of human mobility through online communication data [12].

Traditional socioeconomic studies how economic activities and their context shape social behaviors, and vice versa [13]. These studies reveal how different behaviors are characteristic of different social strata. For instance, income groups feature characteristic patterns of behavior that distinguish them from each other in terms of culture, beliefs, health, and education [14,15,16,17,18,19].

The underlying structure of a social system conditions the behaviors of its members [20]. Similarly wealth also conditions with respect to spaces of mutual exposure and collective learning [21]. Previous studies have shown that income segregation in urban areas determines the places people visit [22,23], the people they interact with, and the topics of conversation they engage in [24]. These analyses show that the segregation of the urban space fragments the social network where information flows and from where behaviors are transmitted and adopted among individuals. Because we learn from imitation, the segregated structure of social networks leads to differentiated social behaviors, including sentiments and emotions [25]. Reinforcing dynamics differentiate behaviors further despite having access to everyone on Internet.

In this paper, we analyze Twitter activity and expose patterns of behavior that are characteristic of different socioeconomic groups and that underlie income prediction tasks. We apply machine learning and information theory methods, including dimensionality reduction techniques, to expose how linguistic and mobility patterns can be used to infer socioeconomic status. More concretely, we analyze the relationship between mobility patterns and hashtag usage with income, as well as the differences between the collective behavior among neighborhoods of different socioeconomic status in terms of the diversity of their interactions.

The paper is organized as follows. Section 2 contains a summary of related studies in the field of income prediction. Section 3 includes a description of the data and the methods we use to collect and analyze it. In Section 4, we present the analysis on mobility and hashtag behavior. In Section 5, we show the structure of the conversational space by means of dimensional reduction. In Section 6, we show signature patterns of socioeconomic groups according to the diversity of their interactions. Finally, we discuss our results in Section 7, and conclude in Section 8.

## 2. Related Work

Methods for inferring demographic information from observations of social behaviors have been recently developed. The availability of social media data combined with traditional sources such as census records enable the observation and analysis of both finer and coarser views of society [4]. Until recently, researchers could only access data from surveys or questionnaires, which by definition are limited in size, scope and frequency, given the difficulties for their deployment and collection. Nowadays, social media data provide researchers with the possibility of observing patterns of behavior which are characteristic of certain demographic groups and therefore enabling the inference of traits from unlabeled individuals.

Twitter is a social media platform where users can post messages and interact with other people. Tweets include metadata with information about the author’s profile, the detected language, as well as the time and location when it was posted. Twitter activity has been analyzed to understand the geography of human sentiments [26], content share networks [6], and dynamics of social influence [27]. It has also been used to advance the understanding of global patterns of human mobility [28], activity [29], and languages [30].

Multiple features have been used in order to predict demographic traits of individuals from the data generated by the usage of multiple types of electronic communication. Socioeconomic status, for instance, has concentrated a great deal of recent attention on the topic. These advances enable a further characterization of the population and prediction of individual attributes such as age [31], occupation [32,33,34,35], political affiliation [36], personality traits [37], and income [32,38]. The properties of Twitter activity and network of followers have also been used to estimate gender and ethnicity [39], unemployment [40], and language [41].

In particular, human mobility patterns are relevant predictors of income. Previous research has shown that the diversity of human mobility is an indicator of economic development across multiple regions [42]. Aggregated data produced by using mobile phones [43,44] and geolocated social media outlets [45] have been crucial in advancing the analysis of human mobility patterns, which are predictable given the regularity of commuting [46] and visitation destinations. Another basis for income prediction is language usage and online content production.

The relationship between income and language has been studied since the early stages of socio-linguistics. At that time, researchers were able to show that social status inferred from someone’s occupation determines the language used [47]. Recently, advances in machine learning take advantage of this social property to build automated classifiers and infer income from behavioral traits [32,33,34]. Gaussian Processes have been applied to predict user income, occupation, and socioeconomic class based on demographics, as well as psycho-linguistic features and standardized job classifications. These technologies map Twitter users to their professional occupations. The high predictive performance has proven this concept with r=0.6 for income prediction, precision of 55% for 9-ways SOC classification, and 82% for binary SES classification. These results further solidify the use of semantic features as proxies to predict individual socioeconomic status.

Furthermore, in a previous work [21], we analyzed the collective topics of conversation coming from neighborhoods of different income in multiple cities around the world. Wealthier neighborhoods tend to discuss lifestyle topics such as travels or leisure, while economically deprived neighborhoods seem to be characterized by other topics of conversation such as sports or TV shows. Second, we noticed that the frequency of visitation between neighborhoods was consistent with the similarity of their topics of conversation. Therefore, neighborhoods that are segregated from one another, such as the case of cities that are segregated by income, tend to also be separated in the space of online conversations. Other studies of urban segregation using geolocated Twitter data confirm that different ethnic groups are less exposed to each other because of segregated residential and travel patterns [22,23].

## 3. Data and Methods

The goal of this paper is to expose patterns of behavior that are characteristic of socioeconomic groups and to show that variations of behavior can be used to derive income predictors. The research question is as follows: Which patterns of Twitter activity are characteristic of different socioeconomic groups and how can we expose them? For this purpose, we downloaded and analyzed Twitter data using statistical, computational, and machine learning methods. We studied multiple aspects of human activity observable from Twitter data. These included mobility patterns, language usage via hashtag adoption, and social interactions via mentions. In this section, we detail the methods used to collect and treat Twitter data, as well as the representation model we created to analyze patterns of behavior from individuals and neighborhoods.

The data have been collected using the Application Programming Interface (API) provided by Twitter for streaming content [48]. The stream API provides around 3% of the overall Twitter activity in real time [49] and over 90% of publicly available tweets with geo-location [50]. The geo-location feature provides precise coordinates of individuals as they post messages. Specifically, we collected over 100M tweets posted by over 2M users, from August 2013 to August 2015, from two European countries. Global statistics show that ~10% of tweets contain hashtags and ~50% of them have at least one mention to another user [51].

Previous studies have analyzed biases in geolocated Twitter users [52,53,54]. In general, Twitter users trend younger, wealthier and urban. However, the under-aged population is underrepresented and the wealth of individuals seems irrelevant in most American cities [21]. These biases can be understood as imbalanced samples, and can be resolved with the corresponding techniques to under-sample over-represented populations. Despite these observations, more recent studies have shown that opinions collected on social media around relevant topics do not differ from the ones one would observe through traditional surveys [55].

In Section 4, we analyze how multiple features of human mobility and online conversations change when conditioned on income. For this purpose, users are characterized with vectors representing either neighborhoods visited or hashtags used. The non-zero elements of the mobility vector represents neighborhoods where the user tweeted from. We assume people’s home locations according to the neighborhood most frequently visited at nighttime during weekdays. The use of these methods is consistent with the procedures followed by other studies relying on mobile phone [56] and Twitter data [46]. We first assign individuals to neighborhoods and then label each user with the average neighborhood income provided by the Census data. The income labels are used to build predictors and characterize the collective activity of the different socioeconomic groups. The non-zero elements of the hashtag vector represents the ones adopted by each user. Hashtags are text labels people use to identify tweets with ongoing events or trends. Hashtags can be used as proxies of collective attention, and their usage has a clear relationship to human distinctive behaviors either in large-scale cultures down to urban life.

We create two independent feature spaces: one representing human mobility and another representing hashtag activity. In both cases, samples represent individuals. In the mobility feature space, the features indicate neighborhoods visited. In the hashtag feature space, each dimension represents whether a given hashtag has been used. We only consider hashtags used by at least five people. We set up a threshold because hashtag usage follows a power law distribution [57]. Most hashtags are used by a single user, while a few of them are used collectively. By doing so, we reduce considerably the number of hashtags and therefore limit the overall dimensionality of the parameter space. Each feature space is then transformed using TF-IDF (Term Frequency-Inverse Document Frequency [58]). This transformation is often used to classify documents based on patterns in their text bodies before using topic models. The underlying assumption behind topic models is that documents that have similar content will tend to also share similarities in their word usage. TF-IDF improves the process of topic discovery by highlighting local information as opposed to globally used terms. Otherwise, very common words or words that appear in single documents would create uninformative signals.

In Section 5, we derive topics of conversation for each user and analyze their overall structure. The topic analysis is generated by means of a *word2vec* model with skip-gram architecture and negative sampling [59]. *Word2vec* is a natural language processing (NLP) technique based on neural networks. The model generates a representation space where pairs of words that are structurally or semantically similar to each other are located in close proximity. This property is due to the architecture behind *word2vec*. The skip-gram architecture predicts the context of words and learns the relationships between words based on their proximity in the text. Negative sampling reduces the number of parameters to train and therefore improves computation time. In this process, words are mapped and embedded onto 50-dimensional vectors. Topics of conversation are then derived by clustering the word co-ocurrence matrix.

Finally, in Section 6 we characterize the collective activity of neighborhoods via interaction vectors. These vectors represent the aggregate behavior of the neighborhoods’ inhabitants. We create mobility and online communication vectors. Mobility vectors aggregate the frequency that individuals from neighborhood *i* visit neighborhood *j*. Online communication is measured via the mentions mechanisms. Mention vectors represent the number of times people from neighborhood *i* mention other people from neighborhood *j*. Unlike the passive information exposure and lesser social involvement reflected by the follower network, the mutual mention network has been shown [60] to better capture the underlying social structure between users.

## 4. Mobility and Hashtag Space

In this section, we discuss properties of mobility patterns and hashtag usage with respect to income. We apply two learning algorithms to the mobility and hashtag feature spaces defined in Section 3. The first algorithm is a Multi-Layer Perceptron (MLP) [61] regression that predicts income as a numeric value. The other one is based on predicting the income quantile using an MLP classifier. We apply them to both mobility and hashtag space. For this purpose, we divide the sample in a training set with 75% of individuals and a test set with the remaining 25% of them. We create multiple samples in order to analyze the performance of the predictors behavior as a random variable. Bootstrapping the performance enables more robust understanding of the prediction quality.

**Figure 1:**
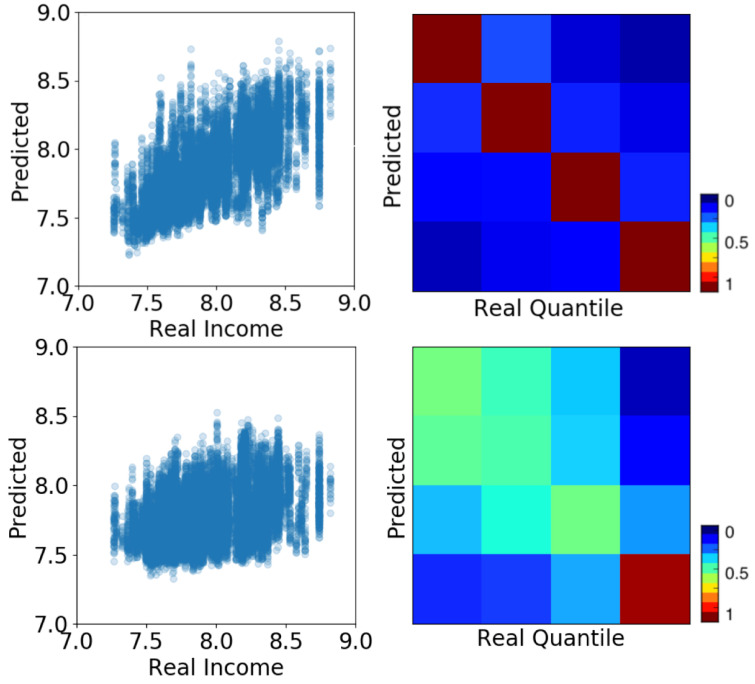
Income prediction based on mobility patterns (**top**) and hashtag usage (**bottom**). Left panels show scatter plots of actual (x-axis) and predicted (y-axis) income using regression. Right panels show the prediction of income quantiles using a classifier. Each quantile represents 25% of the population sorted by income (from left to right, and top to bottom). The matrix elements quantify the number of guesses for each quantile pair (confusion matrix). Scale in figure.

**Figure 2:**
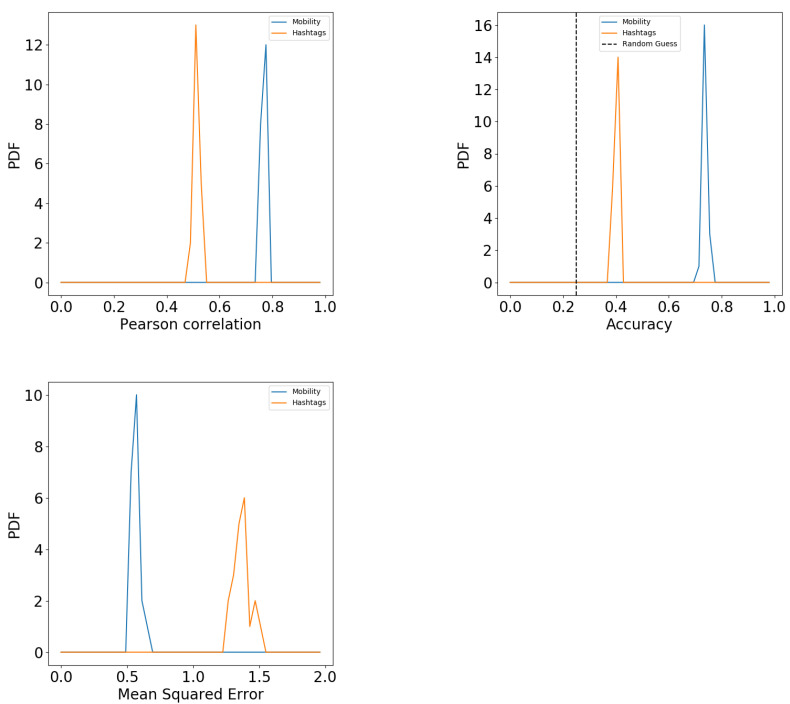
**Top left**: Pearson correlation between predicted and real income values using Regression. **Top right**: Accuracy of the classifier used to predict income quantiles. Dashed line shows random guess. **Bottom**: Mean square error of the income quantile prediction using classifiers. Units are income quantiles. In all panels, distribution represent bootstrapping results. Colors indicate mobility or hashtag usage feature space.

Figure 1 shows the results of the prediction both numerically (left panels) and categorically (right panels). The top panels show the results of the human mobility feature space. The bottom panels show results of hashtag usage feature space. The results of the regressor are shown in the left panels as scatter plots showing the predicted values (y-axis) against the real ones (x-axis). The scatter plots show the overlapped results of the multiple samples we create to bootstrap the algorithm’s performance. For each sample, we calculate the Pearson correlation. We present the distribution of these correlations in Figure 2 (top left). The correlations are high with an average of r=0.8 for the mobility feature space and r=0.55 for the hashtag one. In both cases, a considerable part of the variance is explained by the algorithm.

The right panels in Figure 1 show the categorical prediction of socioeconomic quantiles rather than the numeric values. Diagonal values correspond to True Positives and off-diagonal values represent errors or miss-classifications. The matrices show a strong diagonal structure indicating a very good prediction quality. In the case of mobility (top matrix) the diagonal is almost perfect. In the case of hashtag (bottom matrix) the results are more diffused. However, the wrong predictions are close to their original values and not homogeneously distributed among quantiles. This indicates that errors are not randomly distributed and that contiguous socioeconomic strata have similar behaviors.

The error structure presented in the classification matrices can be interpreted as a behavioral distance among individuals from different quantiles of the income distribution (right panels in Figure 1). The more misclassifications among individuals of different income quantile, the closer their behavior. Previous studies also use similar prediction accuracy as a proxy of cultural distance [16]. In this case, the upper right and bottom left corners of the matrix are colored by darkest blue, showing the least amount of error. This means that the top and bottom socioeconomic quantiles have the most differentiated behavior and therefore are easier to classify.

The bootstrap of the prediction accuracy is shown in Figure 2 (top right). Both hashtag (orange) and mobility (blue) are significantly higher than the error guess (dashed line). Therefore, a considerable part of the variance of hashtag usage and mobility patterns are explained by income. Another way of measuring the prediction quality is through the Mean Square Error (MSE). As quantile labels are also numerical, we can estimate the error of the prediction using the average euclidean distance between the real and predicted value. The MSE in Figure 2 (bottom) shows that the while prediction errors are lowest when using mobility features, the ones obtained from hashtags are still low—with an error below 1.5 quantile difference. Studies based on semantic features and topics of conversation report similar predictive performance [62].

The relationship of mobility and communication has been observed using mobile phones [63] and social media data [24]. People tend to communicate with places they have already visited. Moreover, patches in the territory that host certain populations are consistent with their geographic communication at multiple scales, from national levels down the suburban granularity [12]. These analyses have shown that income fragments the human mobility patterns in cities due to neighborhood segregation and therefore also affects the way people interact with each other both offline and online [21]. Furthermore, previous studies have already hinted toward the existing correlation between the socioeconomic status of people and the diversity of locations they visit. Indeed, as previously pointed out [44], high SES users tend to have patterns of mobility that are more diverse than the ones observed among low SES users, which in turn leads to the lower predictability of their whereabouts. These results may relate to previous work [64,65], which explains this trend by means of the positive payoff between commuting farther for better jobs, while keeping better housing conditions. This in turn also explains why mobility might be used as an indicative predictor of an individual’s socioeconomic status.

## 5. Topic Analysis

Conversational patterns differ by people’s income. Following the methodology developed in a previous study [62], we characterized users by a probability distribution over a set of predefined topics rather than a frequency distribution over all the words of a given dictionary. Topics represent a latent word space such that certain words create topics and users sample words from the topics they talk about. The topic analysis creates a new space of reduced dimensions that represent new features. These new feature can input the classic algorithms used to predict a user’s income based on their tweets.

The topic analysis begins by training a *word2vec* model with the skip-gram architecture and negative sampling on a given collection of tweets [59]. The skip-gram architecture predicts the context of words given their location in sentences. It learns the relationships between words under the assumption that their proximity in the text is not independent of their meaning. The negative sampling method is used for reducing the number of parameters to be inferred in the network. These methods are commonly used for natural language processing. During this process, words are mapped onto a 50-dimensional vector. The words that co-occur in the same tweet will be embedded in vectors that are in proximity to each other. The co-occurring words becomes the basis for deriving conversational topics.

**Figure 3:**
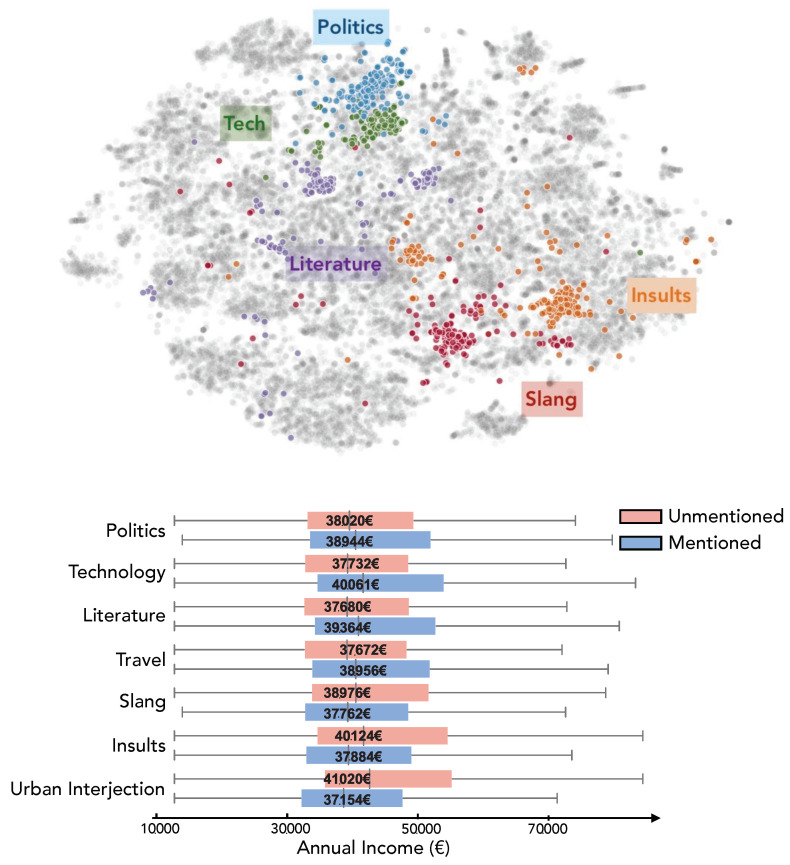
Topic model analysis of tweets per individual. Top panel: 2D visualization (t-SNE) of the embedding space obtained from applying *word2vec* on skip-grams and negative sampling. Colors correspond to topics obtained via clustering. Bottom panel shows income distributions of those who talk about the topics identified.

In Figure 3 (top panel), we show a 2-dimensional representation of the embedding space using t-SNE for visualization. Words are represented by dots, and their proximity is not encoded by the euclidean distance but rather by the cosine similarity value existing between pairs of word vectors. By running a spectral clustering algorithm on the word-to-word similarity matrix and setting negative similarity values to null we derived a prefixed number *d* (here d=100) of clusters of words or topics grouping similar words. These topics were then manually labeled based on which words they contained. In the visualization, some topics have been colored with distinct colors and labeled after manual inspection.

We obtained a distribution of topical interest for users by computing the frequency of use of a given topic over a user’s tweets. The individual vectors show the normalized usage frequency of words from each topic. These vectors coarsely represented users’ syntax and interests and can be used to cluster individuals based on areas of interest. More importantly, we can observe differences among topic vectors based on people’s income. In Figure 3 (bottom panel) we show the income distribution of the individuals who mentioned (or not) a given topic. Individuals that talk about politics, technology, literature and travel have in average higher income than users who did not talk about these issues. Analogously, individuals that used slang, insults or urban interjection had a significantly lower income than the population who didn’t use these words.

## 6. Diversity

We also characterize the diversity of collective activity. For this purpose, we create mobility and communication vectors by neighborhood as explained in Section 3. These vectors represent the aggregate behavior of the individuals who reside there. Mobility vectors aggregate the number of times people from neighborhood *i* visit neighborhood *j*. Online communication is measured via mentions and represent the number of times people from neighborhood *i* mentions other people from neighborhood *j* in their tweets.

We measure the diversity of mobility and communications per neighborhood by quantifying the entropy of the collective behavior vectors. Before calculating the entropy we normalize the vectors by their sum, such that they can be defined as probability density functions. We then calculate the entropy of these distributions and divide it by the hypothetical entropy of the uniform distribution which represents the maximum possible value that the entropy function can attain. Therefore, neighborhoods whose entropy is close to 1 have the most diverse patterns of visitation and interaction online, while neighborhoods whose entropy are close to zero have the least diverse patterns of behavior.

**Figure 4:**
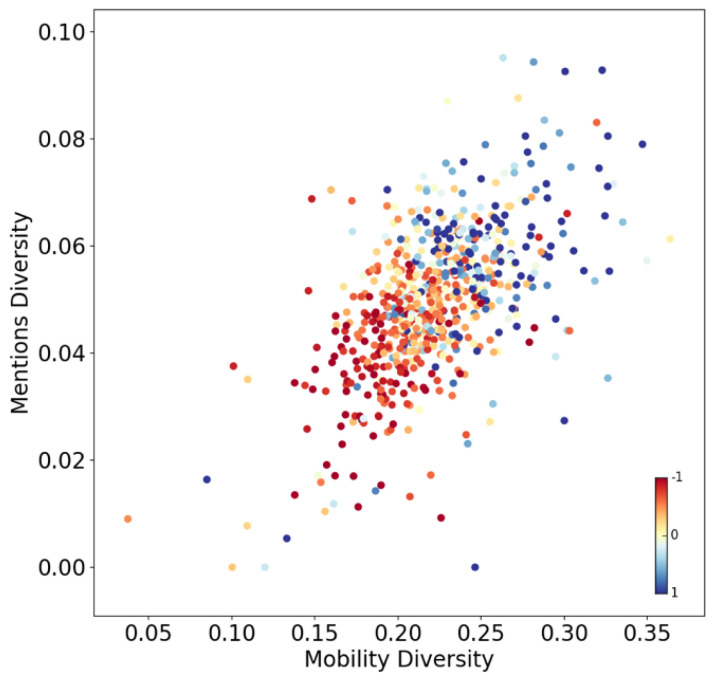
Diversity of collective behaviors in terms of urban mobility and online communication patterns. Dots represent neighborhoods colored by income (from red to blue). The x-axis represents the entropy of mobility vectors aggregated by neighborhoods. The y-axis represents the entropy of Twitter mention vectors aggregated by neighborhoods. Scale in figure. Units represent the number of standard deviations from the mean (centered at zero).

In Figure 4, we present a scatter plot where dots represent neighborhoods colored by income (from red to blue). The x-axis shows the entropy of the mobility vectors and the y-axis shows the entropy of the mentions vectors. There is a direct relationship between the entropy of both vectors. Diverse urban exploration is consistent with diverse online communications (r = 0.57). Moreover, a clear separation of behavior by income is manifested. The diversity for both types of behaviors is consistent with the neighborhood income. Wealthier neighborhoods are consistently more diverse than poorer neighborhoods both in terms of mobility (r = 0.46) and mentions (r = 0.35).

The diversity of social exploration is closely related with the diversity of the information people are exposed to. Those who receive information from multiple sources are more likely to find better opportunities than those who receive information from fewer sources. Therefore, while diverse neighborhoods are also richer, they might be richer precisely because they are diverse. Previous work shows that the diversity of urban exploration is consistent with income and age [66,67]. Our results show that it is also consistent with the diversity of online interactions.

While physical exploration requires resources, online exploration in principle should be considerably less costly. However, the patterns from both offline and online world are remarkably similar. This result is further explored in a previous study [24], where multiple cities are compared and consistent results are obtained from multiple sources of data, including shopping and credit cards. Despite having new methods to interact with one another, people continue to mainly interact with those from their offline lives and behave in similar manners.

In another work [12], we show that people’s mobility and communication patterns online create geographical patches that are preset at multiple scales of observation, ranging from sub-urban areas, up to large national regions. The multiscale nature of these regions arise from the structure of weak and strong ties [5]. While strong ties are local and remain in a radius of 5km approximately, weaker ties span across larger scales, are more diverse and connect distant areas. Previous analysis of social networks show that those long range connections, which are responsible for the spread of information across the whole system, are also unequally distributed among the different income groups.

## 7. Discussion

Inferring the socioeconomic status (SES) of individuals is an important milestone in the development of tools aimed at informing policy makers on how to best curb social problems like income inequality, segregation, and poverty. While nationwide censuses are meant to provide such information, their costs make their collection rather infrequent. Social media analysis on the other hand provides alternative sources of information. Here we provided a general overview on how this can be performed by predicting individual SES based on linguistics and mobility patterns. We showed some of the key patterns that differentiate the behavior of people belonging to different socioeconomic groups.

In order to provide a complete understanding of social context and behaviors, further research should not be bounded to the sole exploration of social media. New approaches relying on widely available satellite imagery and mobile phone data are also proving themselves to be instrumental in capturing part of the inherent dynamics involved in these phenomena, capturing interesting behaviors that had remained hitherto unseen [68,69]. The information provided by these innovative approaches needs, however, to be dealt cautiously. More in-depth studies about the implicit biases underlying these models are necessary before they can be deployed.

The results presented in this paper provide a clear reflection on how complex societal phenomena such as polarization and segregation affect the way we use and interact with social media, which could in turn be used to better understand these social processes. Recent studies indicate that the differentiation of behaviors and physical segregation are deeply intertwined given the reinforcing dynamics of collective learning. The results of these nonlinear processes are reflected in the feature space as unstructured patterns of information that algorithms can use to infer demographic information.

While standardized metrics and measurements are necessary for achieving effective planning, they reduce the description of social and natural systems down to levels that are legible by the policy maker [70]. In some cases, that reduction removes details that are fundamental for the healthy functioning of the system such as relationships and elements that contribute in the background to the stability of the system [1]. The new data sources enable a finer observation of the complexity and varieties of social behaviors and relationships which opens the opportunity for creating plans that are adequate to the complexity of the phenomenon. Being able to observe social behavior at finer granularity brings the mental map closer to reality and increases the amount of available and relevant information to design effective interventions and decision-making processes.

## 8. Conclusions

In summary, we aimed to characterize multiple patterns of Twitter user activity that are related to people’s to show how Twitter user activity differed from user to user when it was conditioned on individual socioeconomic status (SES) differentiate behaviors across multiple social strata and are behind income prediction tasks. In particular, we showed that (1) human mobility is a better predictor of income than hashtag usage, which either way explain a large part of the variance; (2) online topics of conversation and collective interest are strongly influenced by socioeconomic status; and (3) wealthy neighborhoods have more diverse interactions and communication patterns than poorer neighborhoods. These results confirm a segregated and differentiated structure of social groups in both physical and virtual space which in turn enables the prediction of their income.

This study presents certain limitations that open space for further research and future work. Some limitations are related to the methods and representativity of the data. First, we assume that the income of individuals corresponds to the neighborhood average. More advanced methods for inferring home locations could improve such assignment. Moreover, combining both traditional surveys with observational data could improve the income assignation for the training and labeled dataset. Second, the behaviors that we derived can be subject to de-contextualization and generalization which can yield oversimplified views of reality and wrong conclusions. Differentiating between emergent patterns and those within our circle of influence is critical to design effective intervention mechanisms and policies.

The inference of socioeconomic status from widely available digital traces holds a large potential for updating census information as well as enriching other data corpuses with socioeconomic information. This in turn opens the door for further studies to address population level correlations of income with language, space, time, or social network. The use of the aforementioned methods is important as it provides new observations on how socioeconomic status shapes the fabric of society and cements further developments in fields ranging from recommendation systems to economic aid allocation.

## Data Availability

Network data will be available upon publication.
